# Normative values of renin and aldosterone in clinically stable preterm neonates

**DOI:** 10.1007/s00467-022-05807-8

**Published:** 2022-11-21

**Authors:** James Haiyang Xu, Erika Bariciak, Mary-Ann Harrison, Margaret Broom, Brigitte Lemyre, Richard J. Webster, Gabrielle Weiler, Jane E. Dahlstrom, Alison Kent

**Affiliations:** 1 Division of Nephrology, Children’s Hospital of Eastern Ontario, Ottawa, Canada; 2 Division of Neonatology, Children’s Hospital of Eastern Ontario and The Ottawa Hospital General Campus, Ottawa, Canada; 3 Clinical Research Unit, Children’s Hospital of Eastern Ontario Research Institute, Ottawa, Canada; 4 Dept of Neonatology, Centenary Hospital for Women and Children, Canberra Hospital, ACT Australia, SYNERGY: Nursing and Midwifery Research Centre, University of Canberra and ACT Health, ACT Canberra, Australia; 5 Dept of Anatomical Pathology, Canberra Hospital, ACT Australia, Australian National University, Canberra, ACT Australia; 6 Department of Pediatrics, University of Rochester, Rochester, NY USA; 7 Australian National University, Canberra, ACT Australia

**Keywords:** Neonates, Prematurity, Normative values, Plasma renin concentration, Serum aldosterone

## Abstract

**Background:**

There is a paucity of literature on the normative levels of plasma renin concentration (PRC) and serum aldosterone (SA) in premature neonates. This study aims to provide normative data on PRC and SA levels in preterm neonates in the first 2 weeks after birth and explore associations with maternal, perinatal, or postnatal factors.

**Methods:**

Neonates born at 26- to 34-week gestation were recruited from two neonatal intensive care units in Canada and Australia. The direct renin assay PRC and SA were analyzed on day 1 and days 14–21 after birth to compare across categorical variables and to produce normative values.

**Results:**

A total of 262 subjects were enrolled from the Canadian (29%) and Australian (71%) sites. The mean gestational age was 30 weeks, with a mean birth weight of 1457 g. The normative values of PRC and SA for neonates born between 26 + 0 and 29 + 6 weeks and 30 + 0 and 34 + 0 weeks of gestation were produced for day 1 and day 14–21 after birth. Both PRC and SA increased from day 1 to day 14–21. The more premature neonates reached a higher PRC on days 14–21 after birth but exhibited lower SA levels on day 1 after birth. When comparing gender, birth weight, and maternal risk factor categories, no statistical differences in PRC or SA were found. A small but significant decrease in PRC, but not SA, was noted for neonates with placental pathology.

**Conclusions:**

This study produced normative values of PRA and SA in clinically stable preterm neonates that can be referenced for use in clinical practice.

**Graphical Abstract:**

**Figure:**
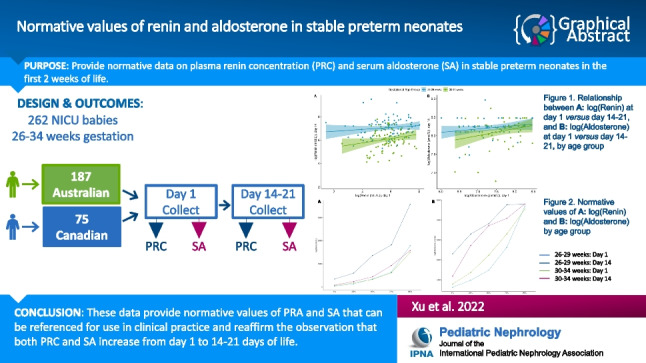
A higher resolution version of the Graphical abstract is available as Supplementary information

**Supplementary information:**

The online version contains supplementary material available at 10.1007/s00467-022-05807-8.

## Introduction

Preterm birth occurs in approximately 1 in 10 births, with an increasing prevalence around the world [1, 2]. Previous studies in both animal models and human subjects have implicated the renin–angiotensin–system (RAS) in the development of lung, cardiovascular, and kidney diseases in those born prematurely [3–9]. Chronic stimulation of the RAS may contribute to hypertension via a sustained increase in vascular resistance, salt retention, and volume expansion, as well as increased inflammation, oxidative stress, fibrosis, and vascular remodeling [10–14].

In neonates, studies of the RAS have demonstrated that renin and angiotensin were markedly elevated compared to adults, with a decreasing trend within the first year of life [15–22]. Some neonatal conditions are associated with changes in RAS response. For example, neonates showed augmentation of the RAS in response to acute changes in blood volume and salt-wasting state [15, 23]. In addition, some preterm neonates with hypertension were found to have elevated plasma renin activity (PRA) and serum aldosterone (SA) levels [24, 25], whereas other studies reported low renin being associated with hypertension [26]. This is complicated by the findings that neonates exhibit physiological partial aldosterone resistance, associated with clinical features of hyponatremia, hyperkalemia, and high urinary sodium loss in the first few days of life [27]. Maternal conditions such as diabetes and altered placental perfusion have been demonstrated to affect neonatal RAS activity and blood pressure as well [28–30].

Assessing renin and aldosterone is useful in the diagnostic and management consideration of neonates with renal hypertension and some electrolyte derangements; however, there is limited literature on the normative levels of PRA and SA in the term neonate, and very little in premature neonates. A prominent renal physiology textbook in the modern era [31] still cites old studies from the 1970s for the normal ranges of PRA [16, 23]. Furthermore, there is currently no study reporting direct plasma renin concentration (PRC) for neonates. PRA is determined by radioimmunological measurement of angiotensin-1 generated from angiotensinogen during in vitro incubation with plasma renin. Not only is direct PRC independent of angiotensinogen levels, but it is also becoming a more widely accepted method of renin measurement than PRA due to its advantages in processing speed, technique standardization, and reproducibility [32, 33].

Establishing normative PRC and SA values in neonates is a valuable addition to the knowledge of clinicians caring for premature neonates. The aim of this study is to provide normative data on PRC and SA levels in preterm neonates in the first 2 weeks after birth and explore whether PRC and SA levels in preterm neonates are associated with maternal, perinatal, or postnatal factors.

## Methods

### Study population

This was a multicenter, prospective cohort study using a convenience sample of neonates admitted to the tertiary level NICUs at the Ottawa Hospital General Campus in Ottawa, Canada, and at the Canberra Hospital in Canberra, Australia. The study at each site was conducted independently as parallel studies, with the Australian cohort funded by a grant from the Canberra Hospital Private Practice Fund and the Canadian site funded by the Physicians Services Incorporated (PSI) Foundation Grant. The study was approved by the Children’s Hospital of Eastern Ontario Ethics Committee (REB 12/06E), the Ottawa Health Science Network Research Ethics Board (REB # 2,011,888-01H), and the ACT Health Human Research Ethics Committee (ETH.11.08.1039). Patients were only entered into this study after free and informed consent was obtained from their parents or guardian. Neonates who delivered between 26 + 0 weeks and 34 + 0 weeks gestational age (GA) and were predicted to be admitted for at least 2 weeks were included in the study. Gestational ages were determined based on dating ultrasound or the last menstrual period. Neonates were stratified into two groups: 26 + 0–29 + 6 weeks gestation and 30 + 0–34 + 6 weeks gestation. The GA cut-off between the groups was to reflect the more immature development of the kidney and increased requirement for respiratory support in the lower GA group. Neonates were excluded if they had known congenital anomalies, including that of the kidneys and the urinary tract, birth asphyxia, known or suspected blood loss at birth, those born to mothers who received teratogenic or nephrotoxic drugs such as an ACE inhibitor, and those with no planned follow-up. For detailed inclusion and exclusion criteria, please refer to Appendix 1. Neonates were withdrawn from the study if they required significant inotropes or postnatal systemic steroids, had greater than 12 h of oliguria or anuria after 24 h after birth, had acute blood loss within the first weeks after birth requiring fluid resuscitation, or had renal vessel thrombosis. Data was collected with respect to maternal medication use and the presence of any maternal or pregnancy comorbidities which could independently affect the PRC and SA levels, such as preexisting essential hypertension requiring medical treatment, hypertension diagnosed during pregnancy with systolic blood pressure > 140 and/or diastolic blood pressure > 80 mmHg, maternal diabetes including gestational diabetes, type I and II diabetes, or fetal growth restriction (< 10th percentile on fetal growth charts).

### Sample collection

Blood for PRC and SA levels was obtained within 6 h and at 14–21 days after birth via venipuncture or arterial line sampling. A blood volume of 1.5 mL was needed if both PRC and SA levels were measured, whereas only 0.75 mL was needed for SA levels only. At the Canadian site, due to protocol restrictions on sample blood volumes for the smallest study subjects, only SA levels were drawn within 6 h of age from those neonates born between 26 + 0–27 + 6 weeks gestation. Day 14–21 both PRC and SA samples were drawn on all subjects. SA was measured using the Siemens Aldosterone Coat-a-Count 125 Iodine radioimmunoassay method. The renin assay was measured on a DiaSorin Advantage automated analyzer. This is a chemiluminescent assay and a direct renin assay. Due to the fact that the assay used to analyze aldosterone at the Canadian site was changed to an unvalidated method during the recruitment phase of the study, SA results obtained during that time period at the Canadian site were discarded. After discarding this result and along with the aforementioned testing protocol restrictions, a total of 14 patients had renin tested on only one day, while a total of 71 patients had aldosterone tested on only one day.

### Placental assessment

The placentas were fixed in 10% buffered formalin and processed using routine laboratory methods. At least two sections of each of the umbilical cord (one toward the maternal end and the other toward the fetal end), extraplacental membranes (including the rupture site), a minimum of three sections of the placental plate, and a decidual shave were submitted for histological assessment. All pathologies were documented, including the weight of the placental disc and any histological changes such as altered villous maturation (fetal and maternal), thrombi, chorangiosis, and decidual vessel vasculopathy. An abnormal placenta was defined as one showing features of altered uteroplacental perfusion. These features were defined as histological evidence of altered villous maturation for the stated gestation and also included the presence of placental infarcts in a preterm placenta or greater than 10% infarction in a term placenta. Placental disc weights were defined as low if they were below the accepted values for gestation [34]. Chorangiosis and the presence of decidual vessel vasculopathy (such as atherosis) were also considered features of altered uteroplacental perfusion.

While the initial reporting pathologist was provided with clinical history, the two pathologists who reviewed the placental pathology for this study did so independently, blinded to the clinical presentation. The two pathologists reviewed the placentas and reported findings separately. If a difference was identified, a consensus was reached.

### Statistical analysis

Central tendency (mean) and dispersion (SD) statistics were produced for continuous variables and frequency distributions for categorical variables. Analyte values where inequalities were reported due to detection limits and the yield of the sample were converted from inequalities to absolute numbers. Clinical and demographic variables were compared between sites using Student *t*-tests or Mann–Whitney *U* tests for continuous variables and Fisher Exact tests for categorical variables. Associations between levels of log(renin) and log(aldosterone) and predictor variables (sex, birthweight, age group, study site, day of testing, and presence of placental pathology) were assessed using linear mixed-effects modeling. The presence of placental pathology was included as a dichotomous variable, wherein the presence of placental infarcts accelerated maturation and evidence of vasculopathy/thrombophilia constituted a positive score. To assess differences in analytes between gestational ages and at different time points, Wilcoxon rank sum tests with continuity corrections were used. Values of PRC and SA were log-transformed to normalize the distribution of these analytes. Normative values (i.e., quantiles), with respective confidence intervals, and all other analyses were calculated using the R statistical software version 4.0.5.

## Results

A total of 75 (29%) and 187 (71%) neonates were enrolled from the Canadian and the Australian site, respectively, and the demographics are shown in Table 1. There were more male (61%) than female neonates in the combined cohort. The mean gestational age at delivery was 30 weeks (standard deviation 2.3 weeks), with 43% (*n* = 114) delivering between 26 + 0 and 29 + 6 weeks and 57% (*n* = 148) between 30 + 0 and 34 + 0 weeks. The mean birth weight was 1457 g (standard deviation 467 g). Four neonates died during the study. The mean blood pressure of the entire cohort was 50.9 ± 7.9 mmHg systolic and 29.8 ± 7.3 mmHg diastolic, without clinically meaningful differences between the Australian and Canadian sites. No subjects required diuretics during the duration of the study.

**Table 1:** Demographics and clinical characteristics (*N* = 262)

Characteristic	Total *N* = 262	Australia *N* = 187	Canada *N* = 75	*P-value*
Male, *n* (%)	158 (60.5)^mv = 1^	105 (56.5)^mv = 1^	53 (70.7)	0.04
Birthweight (grams)				0.01
Mean (SD)	1456.5 ± 458.0	1500.5 ± 476.9	1346.9 ± 388.8	
Median (IQR)	1400.0 (1114.0, 1757.5)	1434.0 (1157.0, 1869.0)	1279.0 (1070.0, 1594.0)	
Gestational age (weeks)				0.53
Mean (SD)	30.3 ± 2.3	30.3 ± 2.3	30.1 ± 2.1	
Median (IQR)	30.6 (28.2, 32.0)	31.0 (28.0, 32.0)	30.0 (28.4, 32.0)	
Gestational age category (weeks), *n* (%)				0.58
26–29 weeks	114 (43.5)	79 (42.2)	35 (46.7)	
30–34 weeks	148 (56.5)	108 (57.8)	40 (53.3)	
Indomethacin use, *n* (%)	15 (5.7)	4 (2.1)	11 (14.7)	< 0.001
Pregnancy conditions, *n* (%)				
Pregnancy induced hypertension	86 (32.8)	62 (33.2)	24 (32.0)	0.89
Essential hypertension	13 (5.0)	9 (4.8)	4 (5.3)	1.00
Diabetes	18 (6.9)	12 (6.4)	6 (8.0)	0.60
Intrauterine growth restriction	15 (5.7)	8 (4.3)	7 (9.3)	0.14
Maternal medication use, *n* (%)				
Steroids	238 (91.9)^mv = 3^	164 (89.1)^mv = 3^	74 (98.7)	0.01
Labetolol	61 (23.3)	40 (21.4)	21 (28.0)	0.26
Nifedipine	24 (9.2)	17 (9.1)	7 (9.3)	1.00
Magnesium sulfate	47 (17.9)	9 (4.8)	38 (50.7)	< 0.001
Any placental pathology, *n* (%)	115 (43.9)	83 (44.4)	32 (42.7)	0.89
APGAR-1 m, mean (SD)	6.4 ± 2.2	6.4 ± 2.1	6.3 ± 2.5	0.97
APGAR-5 m, mean (SD)	8.0 ± 1.4	8.1 ± 1.3	7.7 ± 1.6	0.08
Baseline Systolic, mean (SD)	50.9 ± 7.9^mv = 4^	51.6 ± 8.2^mv = 1^	49.1 ± 6.9^mv = 3^	0.03
Baseline Diastolic, mean (SD)	29.8 ± 7.3^mv = 4^	30.4 ± 7.3^mv = 1^	28.2 ± 7.0^mv = 3^	0.02

*m.v.,* missing values

During data processing and inspection, a batch effect was detected, where the SA results during a portion of time at the Canadian site were beyond the expected range. It was determined that an unvalidated assay was used during this period, and we decided to remove these 163 observations to ensure data quality. In addition, there are 2 (0.86%) and 19 (10%) measurements above detection limits for PRC on day 1 and day 14–21, respectively, and there are 24 and 87 measurements above detection limits for SA on day 1 and day 14–21, respectively.

**Fig. 1:**
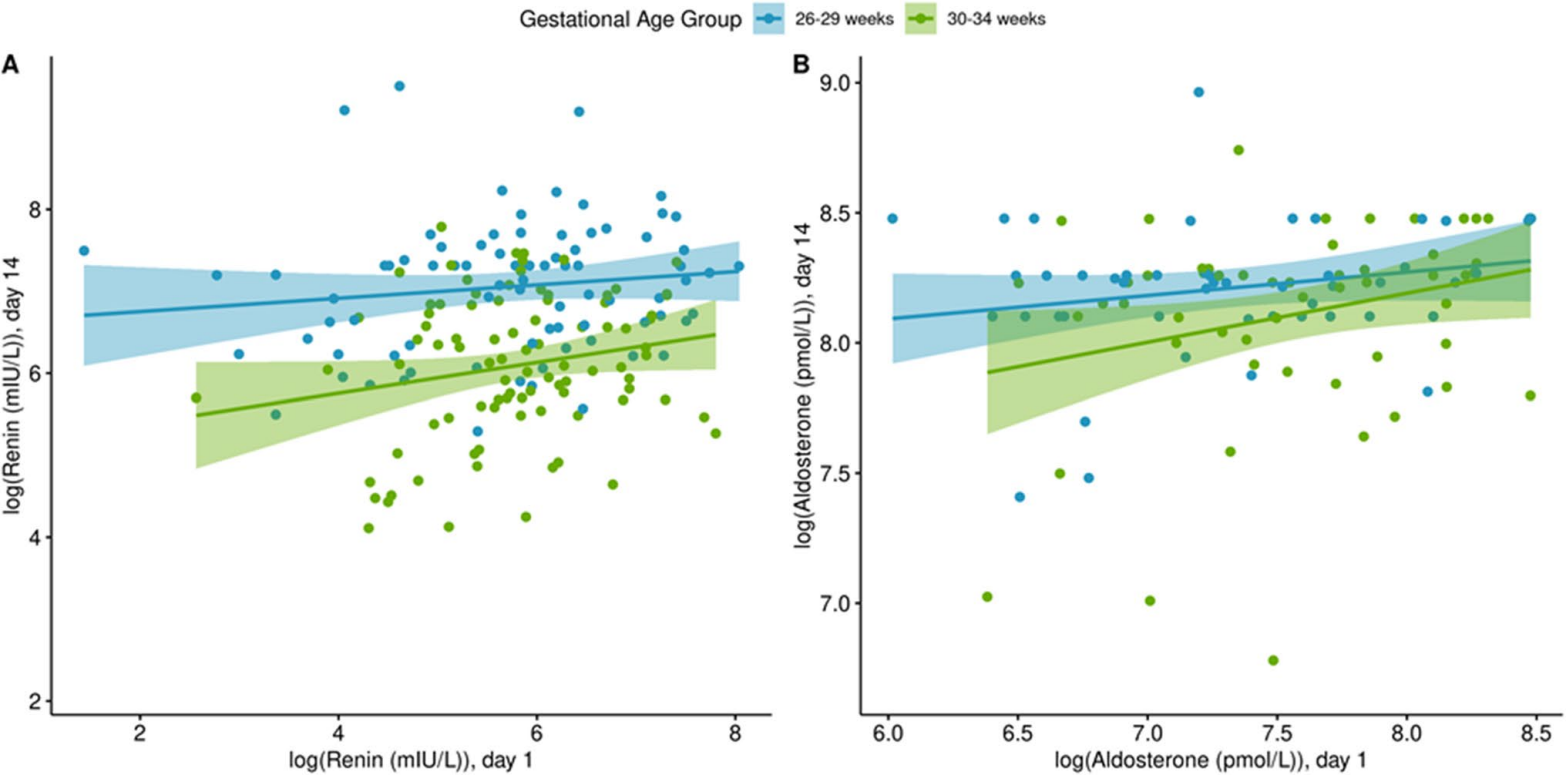
Scatterplot of the relationship between log(renin) and log(aldosterone) at day 1 vs. day 14–21 by gestational age group. A log(renin) day 1, *n* = 230; day 14–21, *n* = 190. B Log(aldosterone) day 1, *n* = 106; day 14–21, *n* = 151); CHEO data from 2013 to 2014 was removed for aldosterone due to a batch effect (day 1, *n* = 37; day 14–21, *n* = 25)

**Fig. 2:**
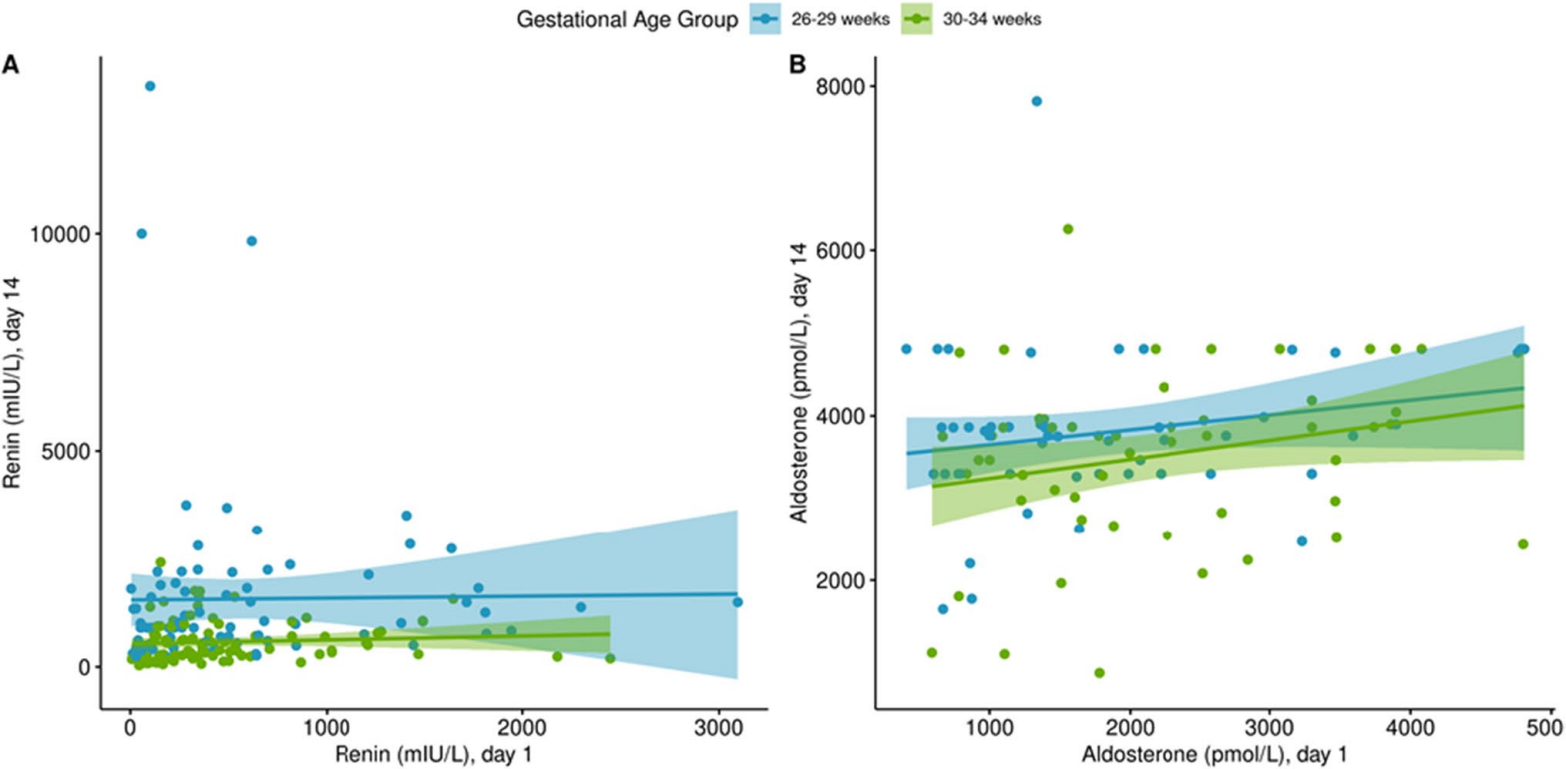
Scatterplot of relationship between renin and aldosterone at day 1 versus day 14–21, by gestational age group. **A** Renin day 1, *n* = 230; day 14–21, *n* = 190. **B** Aldosterone, day 1, *n* = 106; day 14–21, *n* = 151); CHEO data from 2013 to 2014 was removed for aldosterone due to a batch effect (day 1, *n* = 37; day 14–21, *n* = 25)

**Fig. 3:**
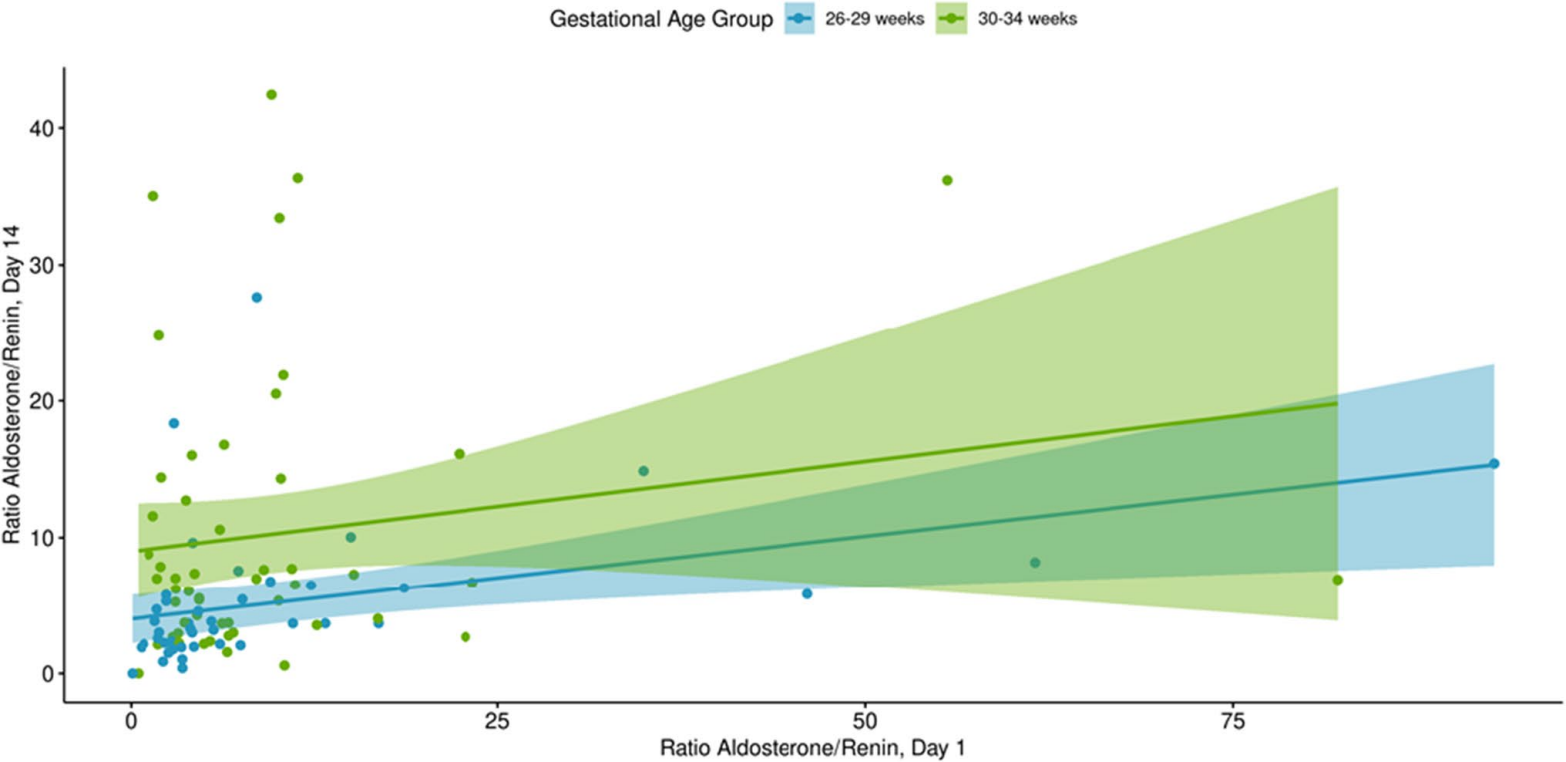
Scatterplot of the relationship between SA/PRC ratio at day 1 vs. day 14–21 by gestational age group. Serum aldosterone (SA)/plasma renin concentration (PRC) ratio, day 1, *n* = 137; day 14–21, *n* = 133. Note: There were 3 (2.1%) cases in which the aldosterone/renin ratio was > 100 at day 1 and 1 (0.7%) case in which the aldosterone/renin ratio was > 100 at day 14–21. These were removed for display purposes

Both PRC and SA levels in neonates, both actual values and operationalized mean logged values, were found to be higher at day 14–21 compared to day 1 after birth (Table 2, Figs. 1 and 2). A similar pattern is observed for the SA/PRC ratio (Fig. 3). There was no statistically significant association found between sex, birth weight, or gestational age with PRC and with SA levels. There was also no difference observed between those with or without maternal risk factors, including gestational hypertension, essential hypertension, insulin-dependent diabetes, gestational diabetes, or fetal growth restriction. A difference was noted in the PRC between neonates from study sites in Canada and Australia. In addition, a small, albeit statistically significant, decrease in PRC was found in neonates with any placental pathology compared to those without. This difference was not noted between these groups for SA levels.

**Table 2:** Multivariable analysis. A Log(renin) (*N* = 419 observations; *N* = 247 patients); B Log(aldosterone) (*N* = 297 observations; *N* = 190 patients)

Variable	Mean ± SD	Adjusted coefficient (95% CI)	*P*-value	Mean ± SD	Adjusted coefficient (95% CI)	*P*-value
Renin				Aldosterone		
Sex			0.53			0.74
Female	6.1 ± 1.2	0 (reference)		7.8 ± 0.9	0 (reference)	
Male	6.1 ± 1.1	0.07 (– 0.14, 0.27)		7.8 ± 0.8	0.03 (– 0.14, 0.27)	
Birthweight (grams)	–	0.00 (0.00, 0.00)	0.07	–	0.00 (0.00, 0.00)	0.50
Site			< 0.001			0.34
Australia	6.2 ± 1.1	0 (reference)		7.8 ± 0.9	0 (reference)	
CHEO	5.8 ± 1.1	– 0.52 (– 0.74, – 0.29)		7.9 ± 0.5	0.15 (– 0.16, 0.47)	
Gestational age category			0.05			0.89
26–29 weeks	6.4 ± 1.2	0 (reference)		7.8 ± 0.9	0 (reference)	
30–34 weeks	5.9 ± 1.0	– 0.28		–	– 0.02 (– 0.30, 0.26)	
Study group			0.99			0.86
Control	6.2 ± 1.2	0 (reference)		7.8 ± 1.0	0 (reference)	
Study	6.0 ± 1.1	0.00 (– 0.23, 0.24)		7.8 ± 0.6	0.02 (– 0.22, 0.26)	
Day of sample collection			< 0.001			< 0.001
Day 1	5.7 ± 1.1	0 (reference)		7.5 ± 0.7	0 (reference)	
Days 14–21	6.5 ± 1.0	0.75 (0.56, 0.95)		8.1 ± 0.9	0.58 (0.44, 0.72)	
Placental pathology			0.01			0.89
No	6.2 ± 1.2	0 (reference)		7.8 ± 1.0	0 (reference)	
Yes	6.0 ± 1.1	– 0.32 (– 0.56, – 0.08)		7.8 ± 0.6	0.02 (– 0.23, 0.26)

The normative values of PRC and SA for neonates are presented in Table 3 for the two gestational age groups. The 50th centile PRC and SA levels increases from day 1 to days 14–21 after birth for both gestational age groups. For PRC days 14–21, the more premature gestational age group had higher normative values. For SA day 1, the more premature gestational age group had lower normative values. In contrast, PRC day 1 and SA days 14–21 had normative values that did not differ by gestational age group.

**Table 3:** Normative values of log(renin) and log(aldosterone) by age group

Condition	Age group	*n*	5%	25%	50%	75%	95%	*P*-value^a^
Renin day 1	All babies	230	49.4	165.2	342.0	631.3	1686.4	0.86
Renin day 1	26–29 weeks	97	37.8	138.0	343.0	641.0	1784.0	
Renin day 1	30–34 weeks	133	56.6	179.0	328.0	610.0	1494.4	
Renin day 14–21	All babies	190	105.4	353.0	752.5	1500.0	2818.6	< 0.001
Renin day 14–21	26–29 weeks	94	341.9	603.5	1358.0	1815.5	3571.0	
Renin day 14–21	30–34 weeks	96	81.5	239.7	437.7	929.0	1574.8	
Aldosterone day 1	All babies	146	667.7	1212.2	1889.5	3300.0	4719.5	0.01
Aldosterone day 1	26–29 weeks	63	633.9	983.5	1485.0	2636.5	4690.0	
Aldosterone day 1	30–34 weeks	83	792.1	1369.5	2240.0	3300.0	4550.2	
Aldosterone day 14–21	All babies	153	1725.0	3265.0	3760.0	4346.0	4808.0	0.16
Aldosterone day 14–21	26–29 weeks	69	2310.8	3300.0	3818.0	4765.0	4808.0	
Aldosterone day 14–21	30–34 weeks	84	1185.3	2722.5	3719.0	4081.5	4808.0	

^*a*^* P*-values were calculated using Mann–Whitney *U* tests

## Discussion

This study is the largest cohort reported to date to establish normative values of PRC and SA concentrations in the first 2–3 weeks after birth for stable premature neonates who were non-oliguric, did not require inotropes, and did not have a deterioration requiring re-intubation. We found that logged PRC levels varied with gestational age, day after birth, and study site (with the day after birth having the largest effect), whereas logged SA levels varied only with the day after birth. These findings are in keeping with similar trends reported in previous studies in the literature. When examining the effects of RAS system-altering maternal comorbid conditions on PRC and SA, we did not find any correlation with these maternal risk factors.

When the sensitive radioimmunoassay methods of measuring PRA and SA using a small quantity of blood became available in the 1970s, there was a cluster of efforts to study the RAS in neonates. The neonatal RAS not only showed heightened activity compared to adults, but it also exhibited gestational age as well as postnatal age-dependent variations. A study in 1972 of 20 neonates first showed that neonate RAS, measured in terms of PRA and renin substrate concentration, was elevated compared to adults [18]. These values rose from 24 h to around 3–6 days after birth but fell at 3–6 weeks to below initial postnatal levels, albeit still higher than adult normal levels established at the time. Similar results were produced in subsequent studies [15, 19, 20], of which some additionally demonstrated increased SA concentration as well in early postnatal life compared to both adults and infants older than 1 month of age. Moreover, several studies specifically examined premature neonates ranging from 29 weeks of gestational age to 36 weeks [21–23]. They illustrated that preterm infants have increased PRA compared to adults, as well as an inverse relationship between PRA and gestational age. A more recent study in 2005, which included neonates born less than 30 weeks gestation, revealed a similar inverse relationship between SA and maturity [35]. These studies suggest that the RAS is stimulated to a much greater extent in premature and term neonates compared to older children and adults, which has implications for both blood pressure and renal electrolyte handling.

The literature on renin levels in premature and term neonates previously had almost all been reported in terms of PRA in ng/mL per time units. Our study measured direct PRC, which is a more efficient and reproducible means of measuring renin activity [32, 33]. There have been no studies that reported direct PRC in the neonatal population since the development of this measurement technique. Thus, a direct comparison with previous literature is not feasible, but it highlights the significance and purpose of this study as a first step in establishing normative PRC values with potential for clinical utility. Our study echoed the findings in other studies that overall PRA levels increase with infant postnatal age within the first two weeks after birth [18]. However, this increase is not as pronounced in the older gestational age group compared to more premature neonates, which is consistent with the previously reported findings of the inverse relationship between PRA and gestational age [21–23], as RAS seems to be stimulated to a higher degree in more premature infants early on in life. This can be explained, at least partly, by the increased renal salt loss and free water loss due to relative aldosterone and ADH insensitivity in the tubules of very premature kidneys [36].

Our study analyzed SA levels instead of plasma aldosterone (PA) levels. We could not find substantial literature that reported on SA levels of premature neonates for comparison. Bourchier [35] reported, in a cohort of 50 neonates with median gestation of 26 weeks (range 24–29), a median PA value of 7300 pmol/L (range 1200–29,000 pmol/L) at day 1 after birth and 8450 pmol/l (1000–30,000 pmol/L) at day 7 after birth. In this study, the 26 + 0–29 + 6-week GA group had a lower 50th centile SA level of 1485 pmol/L (5th centile 634 pmol/L ~ 95th centile 4690 pmol/L) on day 1 after birth and 3818 pmol/L (5th centile 2310 pmol/L ~ 95th centile 4808 pmol/L) on days 14–21 after birth. In previous literature, SA levels were found to be lower than those in the plasma by a median of 50% (41–75%) in one study [37] and by a median of 75% (37–144%) in another [38]. In addition to the variation between serum and plasma aldosterone levels, the discrepancy with our findings compared to Bourchier’s may be due to our study subjects being born at a slightly older gestational age and being less ill since those babies with significant inotrope use were excluded. Studies had previously shown higher PRA and SA trends in more premature neonates and infants with heart failure [8]. On the other hand, for gestational age 30 + 0–34 + 0 weeks, the 50th centile SA level in this study—2240 pmol/L at day 1 after birth and 3719 pmol/L at days 14–21 after birth—is comparable to another smaller group [25] of 15 infants with hypertension and median gestational age of 34 weeks, whose median SA level was 3166 pmol/l (range 704–12,104 pmol/l).

To capture the potential effect of maternal risk factors on neonatal RAS activity, we also separately explored placental pathology and infants whose mothers had gestational hypertension, essential hypertension, insulin-dependent diabetes, or fetal growth restriction. Maternal factors that alter fetoplacental hemodynamics have been shown to affect neonatal RAS. One study showed that PRA was significantly increased in neonates born by vaginal delivery or pregnancy complicated with hypertension compared to elective Caesarean section [28]. In our study, maternal risk factors did not influence the trend in SA and PRC levels. In addition, the presence of placental pathology, including placental infarcts, accelerated maturation, and evidence of vasculopathy/thrombophilia led to no significant difference in SA levels in our study. This contradicts the previously observed clinical effects and postulated mechanisms [26–29]. This may be explained by the relatively small number of neonates in the maternal risk factor group, and because all neonates are premature in this study, the effect of prematurity itself on neonatal RAS might have been much larger and therefore masked the effect of maternal risk factors.

There are important clinical implications for the measurement of PRC and SA levels in neonates. PRA can be used to distinguish between volume-dependent hypertension (suppressed renin) and hyper-reninemic hypertension, which can affect hypertension management [31]. Previous literature recommends screening neonates with sodium and potassium derangements for PRA but not for hypertension [39, 40]. An older retrospective study in 1978 [24] on 17 premature neonates with hypertension showed elevated PRA levels in 11 subjects. They noted potential confounding neonatal conditions previously associated with elevated PRA, including neonatal hypotension, respiratory distress syndrome, kidney failure, and severe lung disease. Seliem et al. [25] reported elevated PRA and SA in 33 and 60% of neonates, respectively, with all-cause hypertension. On the contrary, a novel finding in a group of 97 hypertensive premature neonates without a definite secondary cause and the genetic cause was that of low, rather than elevated, PRA levels along with normal serum sodium, potassium, and SA [26]. Interestingly, the authors of this study noted that spironolactone monotherapy, when used to treat hypertension in these low-renin premature neonates, achieved greater success compared to other antihypertensive agents [26]. However, the source of “normal” ranges for PRA and SA in this study was unclear—there were many “N/A”s, or no range provided, for the normal range for SA and PRA in premature infants at many of the laboratories where the testing was done. Two of the cited laboratories provided a normal range for SA in premature infants but had different levels for different gestational groups at different days of life that were not comparable. Only one lab had a reference range for PRA that did not specify the gestational age included, and the study did not cite the source of the commercial laboratory’s reference values. Similar results were echoed in a case series of hypertensive premature neonates with elevated SA and low PRA, where hypertension did not respond to calcium channel blockers and loop diuretics but did respond to aldactazide [41]. The authors of this study reported that there was limited information on the normal range for PRA based on gestational age and day of life. In fact, they used the values cited in the aforementioned study by Jenkins et al. as their “normal” range [26]. While commercial reference laboratories are required to have quality controls, it is unclear if these controls are from preterm infants or if these labs recruit clinically stable preterm infants from the NICU to produce the reference ranges, as we did in our study. It is also unclear whether these were extrapolated from term infants or adult data, and if so, how were they extrapolated. These gaps in the literature highlight the importance of establishing normative PRC and SA levels in premature neonates based on gestational age in percentiles. This may not only potentially inform the pathophysiology of disease states but also help to guide management decisions and potentially identify neonates at risk for developing hypertension, enabling close outpatient blood pressure monitoring.

This study has several limitations. First, there were some differences in baseline characteristics between the Canadian and Australian sites, suggesting potential underlying confounding factors that might have impacted the combined normative percentile values in this study. For example, maternal magnesium sulfate use was higher in the Canadian site (5% vs. 51%), which could alter maternal BP and thus placental perfusion in the perinatal period. As previous literature has suggested, changes in perinatal fetoplacental hemodynamics could impact neonatal RAS [29, 30]. In addition, there was more indomethacin use in the Canadian site (15% vs. 2%). Indomethacin is known to suppress both PRA and SA in human and animal studies, likely mediated by its inhibitory effect on prostaglandins and, in turn, renal renin production [42–45]. Indomethacin is used in neonates for the treatment of a hemodynamically significant patent ductus arteriosus (PDA), which can limit perfusion to the kidneys and would be expected to increase both the PRC and SA levels. The difference in indomethacin exposure and the presumed presence of a PDA between the two sites could potentially create differences in PRC and SA levels. However, the number of subjects affected by this difference is relatively small and likely would not contribute significantly to the overall results. Thus, we would still suggest the combined data be used for practical purposes. Second, a significant portion of SA data from the Canadian site was discarded, resulting in reduced cases. Another potential confounding factor that was not captured during data collection but may contribute to variability in the results is the change in volume status and serum electrolytes related to diuretics, total fluid intake, and total parenteral nutrition compositions. Although the approach to managing fluids and electrolytes in preterm neonates likely did not vary greatly between these two comparable tertiary-level NICUs and diuretics are not commonly used in the first 2–3 weeks of life in preterm neonates, these are clinical parameters that are closely linked to the RAS axis that could potentially influence the value of PRA and SA levels detected on the day of sample collection. Variability in the management of preterm neonates between NICUs is universal and reflects real-world circumstances, potentially allowing for more generalizability of these results. Given these limitations, future studies could include greater sample sizes and expand the number of study sites with more diverse assay methods.

In summary, this study is the largest cohort to date to study RAS and the normative values of PRC and SA in the clinically stable preterm neonate at various gestational and postnatal ages. It also reaffirmed the observation from older studies that both PRC and SA increase from day 1 to 14 days after birth and that the more premature neonates reached a higher PRC in the first weeks after birth. Contrary to previous reports, however, the more premature neonates exhibited lower SA levels at day 1 after birth compared to those born less premature. When comparing gender, birth weight, and maternal risk factor categories, no statistical differences in PRC or SA were found.

## Supplementary Information

Below is the link to the electronic supplementary material.

Graphical Abstract (PPTX 302 KB)

Supplementary file2 (DOCX 38 KB)
