# Supplementary material for: Normative values of renin and aldosterone in clinically stable preterm neonates
Source: Pediatr Nephrol. 2022 Nov 21;38(6):1877–86. doi: 10.1007/s00467-022-05807-8 (PMC10154272; doi:10.1007/s00467-022-05807-8)
Supplement: Supplementary file 1 — Graphical Abstract (PPTX 302 KB) [file 467_2022_5807_MOESM1_ESM.pptx]

## Slide 1
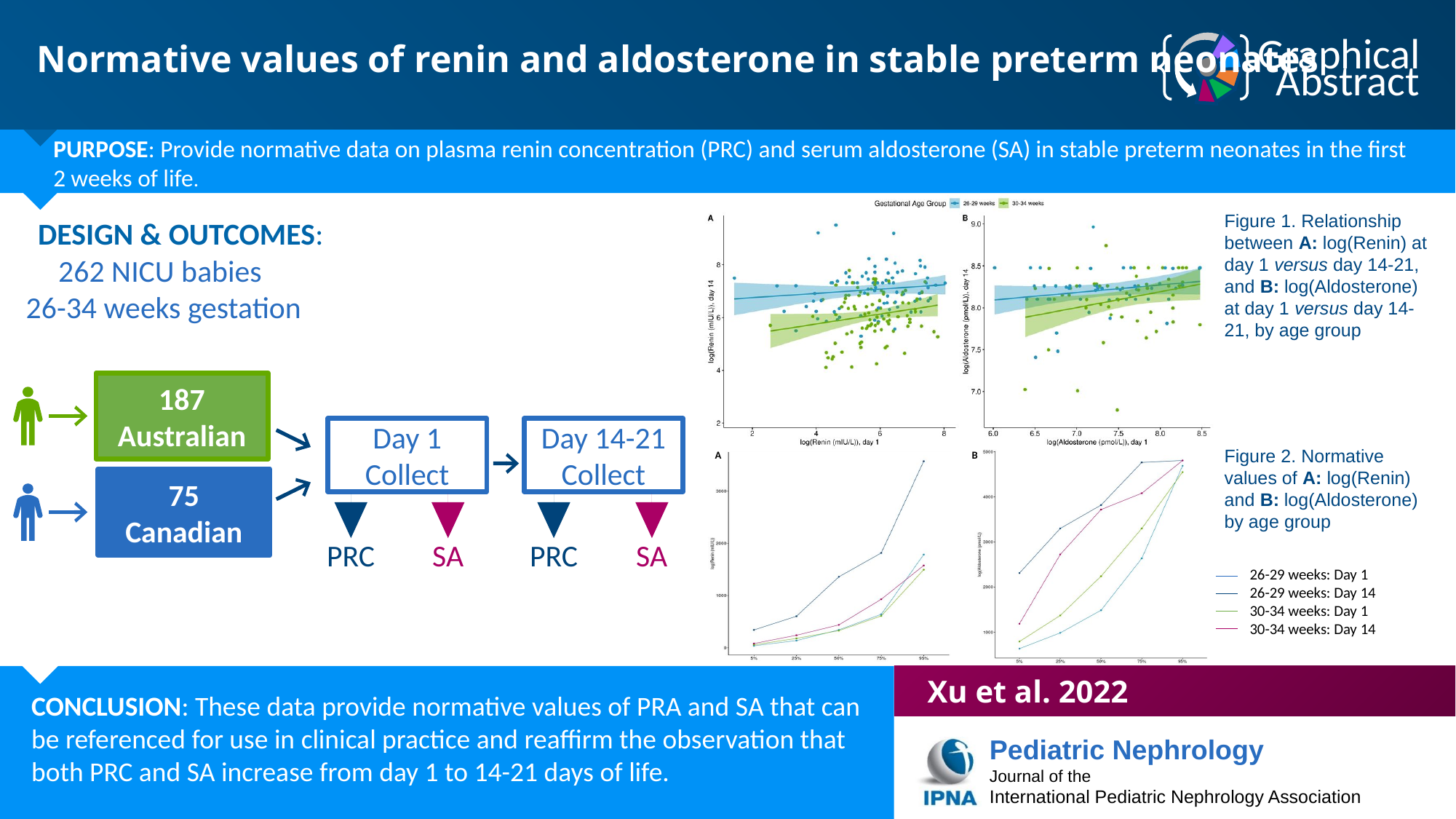

Normative values of renin and aldosterone in stable preterm neonates
PURPOSE: Provide normative data on plasma renin concentration (PRC) and serum aldosterone (SA) in stable preterm neonates in the first 2 weeks of life.
Figure 1. Relationship between A: log(Renin) at day 1 versus day 14-21, and B: log(Aldosterone) at day 1 versus day 14-21, by age group
DESIGN & OUTCOMES:
262 NICU babies
26-34 weeks gestation
187 Australian
Day 1 Collect
Day 14-21 Collect
Figure 2. Normative values of A: log(Renin) and B: log(Aldosterone) by age group
A
B
75 Canadian
PRC
SA
PRC
SA
26-29 weeks: Day 1
26-29 weeks: Day 14
30-34 weeks: Day 1
30-34 weeks: Day 14
Xu et al. 2022
CONCLUSION: These data provide normative values of PRA and SA that can be referenced for use in clinical practice and reaffirm the observation that both PRC and SA increase from day 1 to 14-21 days of life.
