# Supplementary material for: Normative values of renin and aldosterone in clinically stable preterm neonates
Source: Pediatr Nephrol. 2022 Nov 21;38(6):1877–86. doi: 10.1007/s00467-022-05807-8 (PMC10154272; doi:10.1007/s00467-022-05807-8)
Supplement: Supplementary file 2 — Supplementary file2 (DOCX 38 KB) [file 467_2022_5807_MOESM2_ESM.docx]

### Supplemental Report A–D. Univariate Analysis^b^ of Median Normative Values of Renin and Aldosterone by Cohort (Babies Born to Mothers with/without Maternal Risk Factors)

A: Median levels of Renin measured on Day 1: There was no evidence of a difference between babies with and mothers with and without Maternal Risk factors (p=0.24).

B: Median levels of Renin measured on Day 14–21: There was no evidence of a difference between babies with and mothers with and without Maternal Risk factors (p=0.10).

C: Median levels of Aldosterone measured on Day 1: There was no evidence of a difference between babies with and mothers with and without Maternal Risk factors (p=0.43).

D: Median levels of Aldosterone measured on Day 14–21: There was no evidence of a difference between babies with and mothers with and without Maternal Risk factors (p=0.19).

^b^: p-values were calculated using Mann–Whitney *U* tests

***Appendix 1:***

Immediate exclusion from enrollment

1. Maternal treatment with drugs known to be teratogenic and cause nephrotoxicity in the fetus (i.e. ACE-Inhibitor).

2. Infants with known congenital anomalies and/or renal anomalies.

3. Infants with known chromosomal anomalies.

4. Infants with severe asphyxia defined as cord pH <7.0, 5 min Apgar <3, and urine positive for blood.

4. Infants with a known or suspected blood loss at birth (i.e. uterine rupture, placental abruption, bleeding placenta previa, or post-natal blood loss in the delivery room).

5. Infants who will not receive follow-up care in Ottawa upon discharge from the NICU (i.e. live out of town).

Criteria for withdrawal from study

1. Infants who require inotropes in the first 48 hours of life, lasting for greater than 48 hrs.

2. Infants who have a significant illness after the first 48 hrs of life, requiring intubation and inotropes.

3. Infants with oliguria (urine output <1 cc/kg/hr) or anuria after the first 24 hrs of life, lasting >12 hrs.

4. Infants with an acute, significant blood loss within the first two weeks of life, requiring fluid resuscitation.

5. Infants diagnosed with a renal vessel thrombosis.

6. Infants requiring treatment with systemic steroids.

7. At the discretion of the attending physician, those infants deemed to be potentially compromised by the removal of an extra 1.5 cc of blood.
